# Hop-and-sink locomotion and escape in benthic copepods under localized suction flow

**DOI:** 10.1093/plankt/fbag080

**Published:** 2026-11-02

**Authors:** Maximilian Kastinger, Stefano Brizzolara, Tommaso Redaelli, Markus Holzner

**Affiliations:** Institute of Hydraulic Engineering and River Research, BOKU University, Am-Brigittenauer-Sporn 3, 1200 Wien, Austria; Department of Mechanical and Aerospace Engineering, Princeton University, 41 Olden St, Princeton, NJ 08544, USA; Institute of Environmental Engineering, Department of Civil, Environmental and Geomatic Engineering, ETH Zürich, Laura-Hezner-Weg 7, 8093 Zürich, Switzerland; Institute of Hydraulic Engineering and River Research, BOKU University, Am-Brigittenauer-Sporn 3, 1200 Wien, Austria

**Keywords:** benthic copepods, hop-and-sink locomotion, escape response, mechanosensation, 3D particle tracking velocimetry

## Abstract

Benthic copepods inhabit complex boundary layers where flow varies rapidly, yet their sensory-motor responses to localized hydrodynamic signals remain poorly understood. We quantified the behavior of the harpacticoid *Tigriopus californicus* under no-flow and localized suction flow using 3D particle tracking velocimetry of both copepod trajectories and the surrounding flow. Copepods frequently entered the water column and maintained a hop-and-sink gait, spending ~40% of time jumping with peak speeds up to 68 mm s^−1^. In the far field (>2 mm from the source), kinematic statistics were indistinguishable from no-flow controls and orientations were consistent with random swimming. In the near field, individuals performed directed escape jumps (mean 80.6 ± 17.9 mm s^−1^; max. 105.7 mm s^−1^). Escape onset occurred at relative speeds (≈ 6 mm s^−1^) and strain rates (≈ 5 s^−1^) characteristic of localized deformation, at distances from the source on the order of the organism size. These results reveal a context-dependent modulation of locomotion that preserves routine hop-and-sink gait under weak flow while enabling rapid evasion near localized disturbances. The findings are consistent with gradient-based mechanosensation, although direct contact with the suction inlet may have contributed to some escapes. Broader generalization will require replication across taxa, life stages and flow regimes.

## INTRODUCTION

Copepods inhabit almost all freshwater and marine environments, serve as key trophic links at the base of marine food webs, and are considered the most abundant metazoans on Earth (Hardy, 1956). By grazing, respiring, migrating vertically and producing fast-sinking fecal pellets, they drive biogeochemical cycles and contribute to the biological carbon pump, influencing global carbon sequestration and climate regulation (Steinberg and Landry, 2017). These small aquatic crustaceans primarily rely on hydromechanical and chemical cues to perceive and respond to their environment. In particular, their ability to detect and selectively react to weak hydrodynamic disturbances is critical for avoiding predation, finding food, detecting mates and navigating complex habitats (Fields and Yen, 1997; Strickler and Balázsi, 2007; Kjellerup and Kiørboe, 2011). Most copepods can sense light and exhibit phototaxis (Ma and Johnson, 2016), while no evidence of infrared sensitivity has been reported (Porter *et al.,* 2017). However, most species are too small to possess image-forming vision, and visual input is relevant only in a few specialized taxa (Kiørboe, 2011).

Copepods display a range of locomotion modes that vary across developmental stages, species and ecological contexts. The primary modes include: (i) passive sinking and drifting, (ii) feeding motions such as station-holding and the generation of feeding currents, (iii) continuous cruising, (iv) hopping or intermittent swimming, and (v) rapid escape jumps (Strickler, 1975; Jiang *et al.,* 2002; Kiørboe, 2011; Michalec *et al.,* 2020). Routine swimming (cruising) typically occurs at ~0.1–100 mm s^−1^ (Strickler, 1977), whereas peak speeds can reach 100–800 mm s^−1^ (~300–1000 body lengths s^−1^) during escape reactions (Buskey *et al.,* 2002; Lenz *et al.,* 2004).

A growing body of research has demonstrated that copepods possess highly sophisticated mechanosensory systems (Yen *et al.,* 2015). In particular, the characteristic paired antennules, bearing arrays of mechanosensory setae, are the primary hydrodynamic sensors for detecting spatiotemporally structured flow cues (Gill and Crips, 1985; Weatherby and Lenz, 2000; Fields *et al.,* 2002). Setae bend under viscous loading, and this mechanical deformation is transduced into electrical signals. Reported lower thresholds include displacements as small as 10 nm, flow speeds as low as 20 μm s^−1^ (Yen *et al.,* 1992), and strain rates down to 0.04 s^−1^ (Tuttle *et al.,* 2019). Copepods may infer the direction and nature of a flow signal by comparing the amplitude and timing of inputs across setal fields and between antennules (Tuttle *et al.,* 2019), although model-based studies predict “blind spots” and ambiguities in resolving the source side under certain orientations and frequencies (Takagi and Hartline, 2018).

Pelagic species such as *Euchaeta rimana*, *Acartia tonsa*, *Oithona plumifera* and *Bestiolina similis* have been extensively studied and shown to detect and exhibit directional responses to hydrodynamic signals generated by predators, prey and mates (Yen and Strickler, 1996; Kiørboe *et al.,* 1999; Jiang and Paffenhöfer, 2008; Tuttle *et al.,* 2019). They infer the source and identity of a signal from its characteristic kinematic features. Prey-induced hydrodynamic flows are typically weak and characterized by converging streamlines and low gradient magnitudes. Hence, prey detection relies on fluid-body relative velocity (slip) at the antennules and subtle deformation signatures. In contrast, predator approaches produce large gradients, including elevated deformation rates, vorticity and strong unsteady accelerations, that trigger escapes (Kiørboe *et al.,* 1999; Kiørboe and Visser, 1999; Redaelli *et al.,* 2026). Mate-induced signals often have distinct spatial and temporal patterns (e.g. persistent trails, oscillatory features of swimming strokes) and are frequently integrated with chemical cues, enabling tracking and localization (Doall *et al.,* 1998; Strickler and Balázsi, 2007).

Several previous studies have examined responses in various copepod species to predator-induced flows, often mimicked with siphon-generated suction flows (e.g. Fields and Yen, 1997; Waggett and Buskey, 2007; Yen *et al.,* 2015). These investigations have primarily focused on identifying threshold values of fluid-body relative velocity and components of the velocity gradient, such as strain and shear rate, that trigger escape reactions (Kiørboe *et al.,* 1999). In contrast, relatively few studies have analyzed locomotion and orientation within such flow fields (Fields, 2010). Moreover, most species studied to date have been epi- or mesopelagic calanoids (Table I).

**Table I:** Summary of key parameters from studies of copepod escape responses to localized suction flow.

Species	*n* _e_	*u* _jump,e_ (mm s^−1^)	*θ* _jump,e_ (°)	*u* _rel,e_ (mm s^−1^)	$\dot{\varepsilon}$ _e_ (s^−1^)	Reference
*Tigriopus californicus*	8	80.6 (6.3)	150.5 (4.7)	6.4 (1.5)	4.7 (0.7)	This study
*Acartia hudsonica*	41	289 (25)	*	*	2.2	Burdick *et al.* (2007)
*Acartia tonsa*	12–64	*	*	0.13	0.19–1.43	Kiørboe *et al.* (1999)
*Calanus finmarchicus*	11	460 (60)	135.7 (12.3)	*	18.7 (3.0)	Yen *et al.* (2015)
*Centropages hamatus*	13	367 (22)	*	*	1.2	Burdick *et al.* (2007)
*Hesperodiaptomus shoshone*	9	220 (33)	112.6 (3.7)	*	15.1 (4.5)	Yen *et al.* (2015)
*Paracalanus parvus*	28	*	*	2.03	6.16	Waggett and Buskey (2007)
*Temora longicornis*	56	244 (14)	*	*	2.7	Burdick *et al.* (2007)
*Temora turbinata*	34	*	*	1.51	3.93	Waggett and Buskey (2007)
*Tortanus discaudatus*	36	343 (29)	*	*	0.34	Burdick *et al.* (2007)

Number of escape events (*n*_e_), jump intensity (*u*_jump,e_), jump orientation relative to the stimulus axis (θ_jump,e_), relative fluid-body velocity (*u*_rel,e_) and strain rate ($\dot{\mathrm{\varepsilon}}$_e_) at initiation of escape. Values are means (SE). An asterisk (^*^) indicates data not reported.

In contrast to pelagic species, far less is known about the sensory responses of benthic copepods to hydrodynamic cues. Living on or within the substrate, they experience complex, spatially and temporally variable flows in turbulent boundary layers (Pepper *et al.,* 2015). Harpacticoids, predominantly bottom-dwelling copepods, are distributed worldwide and constitute the second-largest group of marine meiofauna (Coull, 1988). They differ from other copepod orders by having relatively short antennules and robust thoracic appendages adapted for crawling and burrowing (Barnes, 1987). At small spatial scales, benthic harpacticoids often display patchy distributions, likely driven by biotic factors such as trophic interactions with food resources (Azovsky and Udalov, 2026). Although primarily benthic, harpacticoids frequently emerge from the substrate into the water column for active migration, employing a jerky hop-and-sink swimming mode (Armonies, 1988; Hwang and Turner, 1995). Such excursions may serve several ecological functions, including reproduction, dispersal, temporary escape from predators or competitors, and relocation to more favorable habitats (Azovsky and Udalov, 2026).

Species of the harpacticoid genus *Tigriopus* predominantly inhabit rocky tide pools in the supralittoral zone, where they exhibit both benthic and free-swimming behaviors. These habitats become isolated from the ocean during low tide and undergo strong fluctuations in physical conditions (Dethier, 1980). Consequently, *Tigriopus* copepods are highly eurytopic, thriving under variable environmental conditions and tolerating wide ranges of temperature and salinity (Ranade, 1957; Matutani, 1961). The species *Tigriopus californicus* (Baker, 1912), distributed along the Pacific coast of North America from Baja California, Mexico, to Alaska, USA, has been extensively studied in laboratory settings (Dethier, 1980). However, while previous research has focused primarily on its physiology, population genetics and responses to environmental stressors (Burton *et al.,* 1979; Willett and Ladner, 2009; Powers *et al.,* 2025), little is known about how *T. californicus* detects and responds to hydrodynamic disturbances.

Considering its primarily benthic habit and morphology distinct from that of pelagic taxa, we hypothesized that *T. californicus* exhibits a lower sensitivity to hydrodynamic disturbances and attenuated escape performance in the water column than those reported for pelagic copepods (Table I). To verify this hypothesis, we quantified the flow field and copepod kinematics with particle tracking velocimetry (PTV), comparing behavior in the absence and presence of needle-induced suction and analysing responses across spatial scales, from weak far-field disturbances to steep velocity gradients near the needle tip. We examined whether (i) routine kinematics in the far field are distinguishable from no-flow controls, (ii) escape onset occurs only above higher hydrodynamic thresholds than those published for pelagic species, and (iii) peak escape speeds are lower than pelagic benchmarks. To our knowledge, this is the first study to quantify locomotion modes and behavioral responses of *T. californicus*, and harpacticoids more broadly, to hydrodynamic cues using a controlled, localized suction flow.

## METHOD

### Copepod collection and maintenance

*Tigriopus californicus* were obtained from a marine aquaculture facility (Stare Polichno, Poland) and shipped in a culture medium composed primarily of artificial seawater supplemented with vitamins and particulate feed. Upon arrival, copepods were maintained in this medium at 8°C, in darkness, without aeration and additional feeding. Under these conditions they remained alive and active for several weeks, consistent with their eurytopic nature. Prior to experiments, individuals along with their culture medium were removed from cold storage and passively warmed to room temperature (18.5–19°C) on the order of a couple of hours. After acclimation, copepods were transferred to the experimental aquarium, containing artificial seawater at a salinity of 28 (practical salinity units). Copepods exhibited normal activity under these conditions for multiple days.

### Experimental setup

The experimental aquarium was constructed from transparent acrylic and had a footprint of 70 × 70 mm with a water depth of 60 mm (Fig. 1a). To minimize disturbances from ambient air currents, the aquarium was covered with a lid. A hypodermic needle (hereafter, “needle”) was positioned vertically at the center, with its tip at mid-depth (30 mm below the water surface). The needle was a 27 G type, with a nominal inner diameter *d*_i_ = 0.21 mm, chosen to be smaller than typical copepod body width. A steady suction flow with volumetric rate *q* = 8 μL s^−1^ was applied via a syringe pump connected to the needle, generating a mean axial velocity in the needle tip of *U*_0_ = 231 mm s^−1^. Test runs commenced only after steady-state flow was established.

**Fig. 1:**
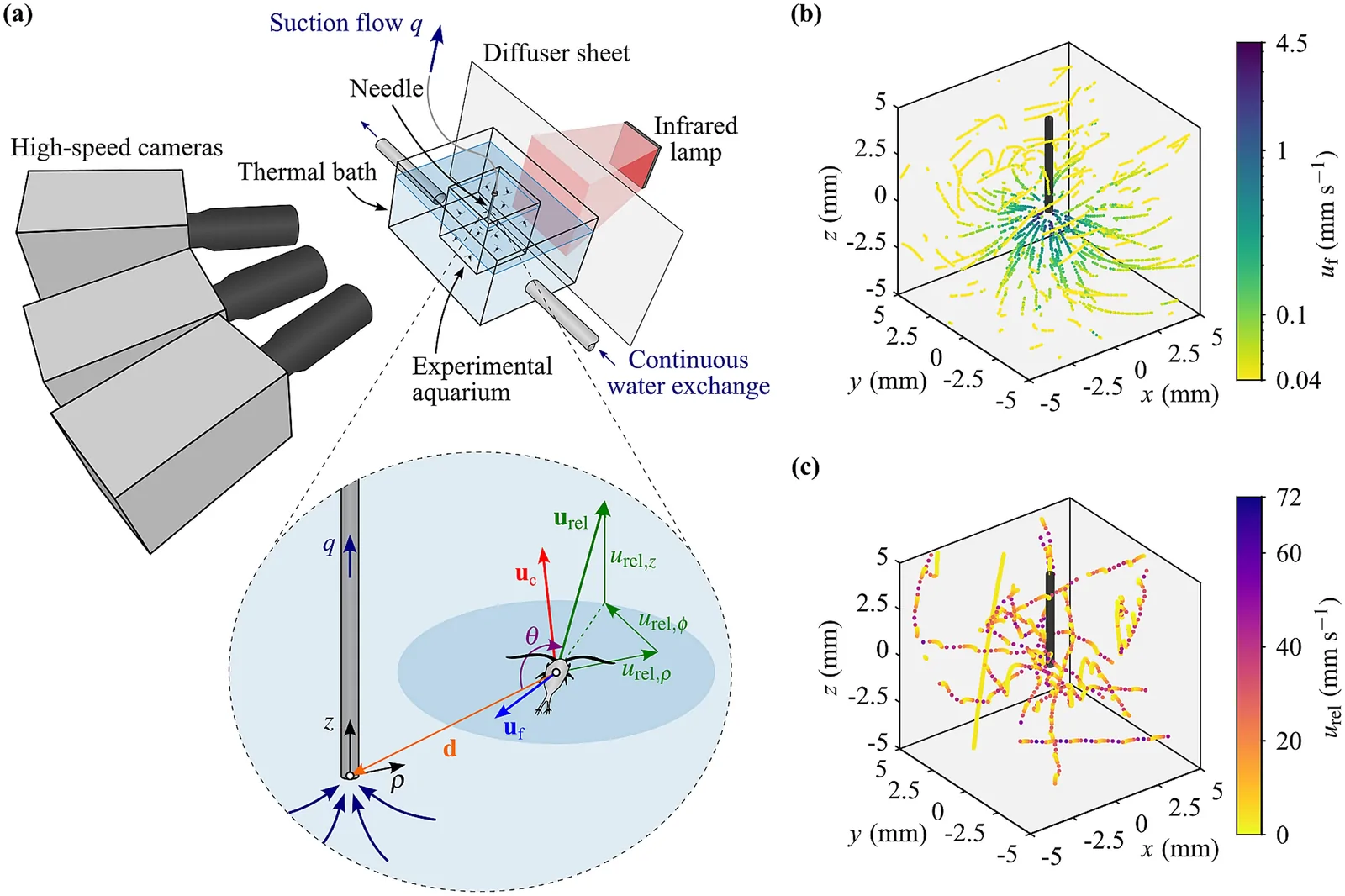
(a) Schematic illustration of the experimental setup and a copepod at distance *d* from the needle tip with instantaneous velocity, **u**_c_, in a local flow, **u**_f_. The angle *θ* denotes movement relative to the copepod-to-tip direction. Example trajectories of (b) tracer particles, and (c) copepods. The coloring in (b) and (c) represents the flow speed, *u*_f_, and the relative velocity magnitude, *u*_rel_, respectively.

All tests were conducted in darkness to standardize illumination and eliminate visual cues, thereby preventing phototactic responses. Imaging illumination was provided from behind the aquarium using an array of high-power infrared diodes (300 W total) (Michalec *et al.,* 2015). To prevent heating and suppress convective flow, the experimental aquarium was submerged within a larger, water-filled container serving as a thermal bath (Fig. 1a). This outer reservoir was continuously circulated with a pump, maintaining the aquarium water at a stable 17–18°C throughout the day.

Experiments were recorded using three synchronized Photron FASTCAM SA5 cameras viewing the measurement volume from different angles through the front walls of the inner aquarium and the surrounding thermal bath (Fig. 1a). With backlit infrared illumination, silhouettes of the needle, seed particles and copepods had sharp edges, and internal features of the partially transparent copepods were discernible. The cameras were fitted with one Nikon 200 mm lens and two Tamron 180 mm macro lenses, set to apertures of *f*/32 and *f*/22, respectively, to maximize depth of field. The measurement volume was a 25 × 25 × 25 mm^3^ cube centered at the needle tip. Image acquisition was carried out at a frame rate of 125 fps, with a resolution of 1024 × 1024 pixels and an 8-bit grayscale depth. Post-processing of the recorded images included background subtraction, Gaussian blur filtering and gray-level threshold-based segmentation, followed by detection of the centroids of segmented flow tracers and copepods. The latter was defined as the area centroid of the copepod silhouette.

We performed three-dimensional PTV of flow tracers and copepods using the open-source software OpenPTV (https://www.openptv.net/). Camera calibration used images of a three-dimensional reference target with 126 control points of known coordinates, uniformly distributed along all three spatial axes (Willneff and Maas, 2000). From these images, both intrinsic and extrinsic camera parameters were determined. The calibrated parameters enabled cross-view correspondences of particle images and reconstruction of three-dimensional centroid positions by forward intersection, treating centroid coordinates as unknowns in an augmented collinearity (projection) model (Maas *et al.,* 1993; Malik *et al.,* 1993). Image sequences were then processed with a multi-view tracking algorithm that combines image- and object-space information to identify and follow the centroids of segmented seed particles and copepods over time (Willneff, 2003). For tracer-based flow characterization, reliable particle tracking was possible down to 0.14 mm from the needle tip. Closer than this, particles disappeared into the needle between frames and could not be tracked further. This proximity limit did not apply to copepod tracking because of their larger size.

To avoid interactions between copepods and tracer particles, we quantified the suction flow and recorded copepod behavior in separate experiments (Fig. 1b and c). Although this precluded time-synchronized flow-behavior pairing, the suction field was steady and reproducible. Therefore, we assigned local flow quantities to instantaneous centroid positions of copepods by spatially registering trajectories to the calibrated coordinate system and sampling the pre-measured flow field. While flow characterization was performed in freshwater, copepods were recorded in seawater. Because fluid density was homogeneous and freshwater–seawater differences in kinematic viscosity at our experimental temperature were minimal, the steady suction flow field was effectively unchanged between experiments with and without copepods.

### Flow characterization

We characterized the flow in freshwater using neutrally buoyant polyethylene tracer particles (Cospheric UVPMS-BR-1.00) with density *ρ*_p_ ≈ 1.00 g cm^−3^ and diameters *d*_p_ = 63–75 μm. Particle velocities were highly heterogeneous across the field, with very slow motion in the far field and rapid accelerations near the needle tip (Fig. 1b). To reduce tracking noise associated with slow-moving particles, we first isolated fast-moving particles using a temporal moving-average filter (window size 45 frames, i.e. 0.36 s), then tracked these at the frame rate of 125 fps. The remaining particles, predominantly in low-velocity regions, were tracked at reduced frame rates of 25, 5 and 1 fps. This multi-frame-rate strategy ensured robust and accurate tracking across regions with varying flow velocities while preserving temporal resolution where needed. Raw position signals were smoothed using a moving third-order polynomial filter, with window sizes of five frames for data recorded at 1 fps and 11 frames for data recorded at 5–125 fps. Because frame rates below 125 fps were used only in low-velocity, slowly varying regions, applying the same frame-window length at 5–125 fps maintained a comparable spatial smoothing scale. Instantaneous velocities were then computed as the first derivative of the fitted polynomial (Lüthi *et al.,* 2005). Filtered data from all frame rates were subsequently combined for further analysis. The dataset of smoothed tracer-particle trajectories is available from Kastinger *et al.* (2026).

Given the cylindrical symmetry of the vertically oriented needle and the absence of nearby boundaries (walls and free surface were outside the measurement volume), the flow field was assumed, and subsequently validated, to be axisymmetric about the needle axis. We defined a cylindrical coordinate system with origin at the needle tip, comprising radial coordinate *ρ*, vertical coordinate *z* and azimuthal angle *ϕ* (Fig. 1a). The corresponding velocity components of the flow field **u**_f_ are *u*_f,*ρ*_, *u*_f,*z*_ and *u*_f,*ϕ*_. Under perfect axisymmetry, *u*_f,*ϕ*_ = 0. The flow speed is therefore *u*_f_ = $\sqrt{u_{\mathrm{f},\rho}^2+{u}_{\mathrm{f},z}^2}$.

Assuming steady flow, instantaneous velocity data were spatiotemporally averaged on a polar grid with radial and angular resolutions Δ*ρ* = 0.3 mm and Δ*φ* = 5°, respectively. At each grid point, the local strain rate was evaluated as $\dot{\varepsilon}$ = $\sqrt{2}$||**S**|| where **S** = $\frac{1}{2}$(**∇u**_f_ +( **∇u**_f_)^T^) is the strain-rate tensor and **∇u**_f_ is the velocity gradient tensor. We computed the vorticity magnitude *ω* = ||**∇ × u**_f_ || and evaluated the second invariant of the velocity gradient tensor, *Q* = $\frac{1}{2}$(||**R**||^2^ − ||**S**||^2^) = $\frac{1}{4}$(*ω*^2^ − $\dot{\varepsilon}$^2^), where **R** = $\frac{1}{2}$(**∇u**_f_ −( **∇u**_f_)^T^) is the vorticity tensor. Regions with *Q* < 0 were classified as strain-dominated and those with *Q* > 0 as rotation-dominated (Hunt *et al.,* 1988; Jeong and Hussain, 1995). Analyses were restricted to a cylindrical observation volume centered on the suction axis, with radial extent *ρ* ≤ 10 mm and vertical extent |*z*| ≤ 10 mm.

The characteristic Reynolds number at the needle tip is Re_0_ = *U*_0_  *d*_i_/*ν*_f_ where *ν*_f_ = 1.05 × 10^−6^ m^2^ s^−1^ is the kinematic viscosity of water at 18°C. With *U*_0_ = 231 mm s^−1^ and *d*_i_ = 0.21 mm we obtain Re_0_ ≈ 46, indicating laminar internal flow and a low to moderate Reynolds number near the tip. The local Reynolds number within the external flow field was approximated by Re_f_ = *u*_f_  *L*/*ν*_f_ ≈ *u*_f_^2^/(*ν*_f_ ||**∇u**_f_ ||), with a characteristic length scale *L* ≈ *u*_f_/||**∇u**_f_ || derived from the local velocity gradient tensor.

The Stokes number, St = *τ*_p_/*τ*_f_, compares the particle relaxation time *τ*_p_ to a characteristic flow timescale *τ*_f_. In the Stokes regime, the relaxation time is *τ*_p_ = *ρ*_p_  *d*_p_^2^/18*μ*_f_, with *μ*_f_ = 1.0 × 10^−3^ Pa s (dynamic viscosity of water at 18°C). For the particle size range used here, this yields *τ*_p_ = 2.2–3.1 × 10^−4^ s. We estimate the flow timescale as *τ*_f_ ≈ 1/$\dot{\varepsilon}$ and find it smallest (0.134 s) at the minimum distance from the needle tip at which tracking was achieved (0.14 mm), where velocity gradients are steepest. Thus, the maximal Stokes number occurs there and is St_max_ ≈ 1.6–2.3 × 10^−3^, well below 0.01. Consequently, tracer particles behaved as passive tracers throughout the observation volume (Tropea *et al.,* 2007).

### Copepod behavioral experiments

For the behavioral study of *T. californicus*, adult males and females were tested together in mixed groups. Prosome length was ~0.5 mm, as estimated from video recordings. No food was present during the test runs. In the suction flow, we conducted 24 test runs, each 2 min in duration and recorded at 125 fps. Four additional runs with the needle present but no flow (**u**_f_ = 0) served as controls.

The observation volume matched that used for flow characterization. Trajectory segments belonging to the same individual were manually glued and validated, allowing a maximum temporal gap of five frames (0.04 s) between detections. Trajectories shorter than 1 s were excluded yielding 615 trajectories for the suction-flow treatment and 90 trajectories for the no-flow treatment for further analysis. Raw position data were smoothed using a moving third-order polynomial filter (window size five frames), and instantaneous velocities were computed as the first derivative of the fitted polynomial (Lüthi *et al.,* 2005). The datasets of smoothed copepod trajectories for both the no-flow and suction-flow conditions are available from Kastinger *et al.* (2026).

For each copepod position, **d** denotes the vector from the copepod centroid to the needle tip, and its magnitude *d* = ||**d**|| is the centroid-to-tip distance (Fig. 1a). In the suction-flow treatment, the copepod’s absolute velocity, **u**_c_, was evaluated relative to the local flow velocity, **u**_f_, computed as the mean flow within a radius *r*_c_ of 0.5 mm (estimated prosome length) centered on the copepod’s instantaneous position. The fluid-body relative velocity (slip) was defined as **u**_rel_ = **u**_c_ − **u**_f_, representing the direction of active motion. Analogous to the flow velocity components, the cylindrical components of **u**_rel_ are *u*_rel,*ρ*_, *u*_rel,*z*_ and *u*_rel,*ϕ*_ (Fig. 1a), and the relative speed is *u*_rel_ = $\sqrt{u_{\mathrm{rel},\rho}^2+{u}_{\mathrm{rel},z}^2+{u}_{\mathrm{rel},\phi}^2}$. A sensitivity analysis varying *r*_c_ from 0.1 to 1.0 mm showed a negligible effect on *u*_rel_. Velocity components projected along **d** were computed as *u*_i,*d*_ = **u**_i_ ⋅$\hat{d}$, where $\hat{d}$ = **d/**||**d**|| is the unit vector pointing from the copepod toward the needle tip, and i ∈ {f, c, rel} denotes flow, copepod or relative velocity. The angle *θ* denotes the copepod’s movement direction relative to **d**, defined by cos*θ* = **u**_rel_ ⋅ **d/**(*u*_rel_  *d*). By this convention, *θ* = 0° and 180° corresponds to movement directly toward and away from the needle tip, respectively. During jumps, copepod body orientation was approximated by the direction of **u**_rel_.

Jumps associated with hopping or escape responses were identified as local maxima in the temporal relative-velocity signal, *u*_rel_(*t*). The magnitude of each peak, *u*_jump_, quantified jump intensity. To minimize false detections from residual noise, only peaks exceeding 10 mm s^−1^, ~4*u*_sink_, were retained, where the mean sinking speed *u*_sink_ = |${\overline{u}}_{\mathrm{rel},z}$| was computed from trajectories exhibiting exclusively downward sinking. Peaks in *u*_rel_(*t*) separated by <0.1 s were merged and classified as a single jump event, with the maximum peak within the merged interval taken as *u*_jump_. This 0.1 s criterion is considerably shorter than typical inter-jump intervals reported for copepods (Kiørboe *et al.,* 1999; Michalec *et al.,* 2017). Jump duration, Δ*t*_jump_, was defined as the time interval between the local minima in *u*_rel_(*t*) immediately preceding and following the peak. Jump distance, *d*_jump_, was obtained by integrating **u**_rel_(*t*) over the jump duration. The separation time between successive jumps, *T*_jump_, was taken as the interval between consecutive velocity peaks. To focus on *T*_jump_ within hopping sequences, we fitted a two-component Gaussian mixture model to the *T*_jump_ distribution and retained values from the cluster with the shorter mean, excluding long inter-jump intervals that spanned sinking phases (i.e. *T*_jump_ > 0.32 s). Unless otherwise noted, summary statistics for all variables are reported as mean ± standard deviation (SD) for comparability with prior literature.

## RESULTS

### Flow characterization

We assessed axial symmetry of the suction flow about the needle axis by comparing the absolute azimuthal velocity component, |*u*_f,*ϕ*_|, to the flow speed, *u*_f_. Over the entire cylindrical observation volume, the ratio of spatially averaged values was 〈|*u*_f,*ϕ*_|〉/〈*u*_f_〉 = 0.22. The maximum local |*u*_f,*ϕ*_| was 0.72 mm s^−1^ at a local *u*_f_ = 3.75 mm s^−1^, yielding a pointwise ratio of 0.19. Because out-of-plane flow speeds (*ϕ*-direction) were only ~20% of in-plane (*ρ*-*z*) magnitudes, we treated the flow as axisymmetric about the needle axis for subsequent analysis.

Quantification of the flow field showed a well-defined directional pattern and a continuous increase in *u*_f_ beneath the needle (*z* < 0) toward the tip, within ~4 mm of the tip (Fig. 2a). Near the tip, the velocity gradient was steep, with *u*_f_ reaching a maximum of 3.75 mm s^−1^. The strain rate, $\dot{\varepsilon}$, likewise increased toward the tip within 4 mm, reaching a maximum of 7.5 s^−1^ (Fig. 2b). In addition to this primary pattern, a weak, upward-directed recirculation with *u*_f_ up to 0.05 mm s^−1^ was observed for *z* > 1 mm in the immediate vicinity of the needle. In the far field, beyond 4 mm from the tip, *u*_f_ remained below 0.09 mm s^−1^. Distance-specific mean experimental flow speed (Fig. 2c) and strain rate (Fig. 2d) within 4 mm of the tip agreed well with a theoretical sink flow.

**Fig. 2:**
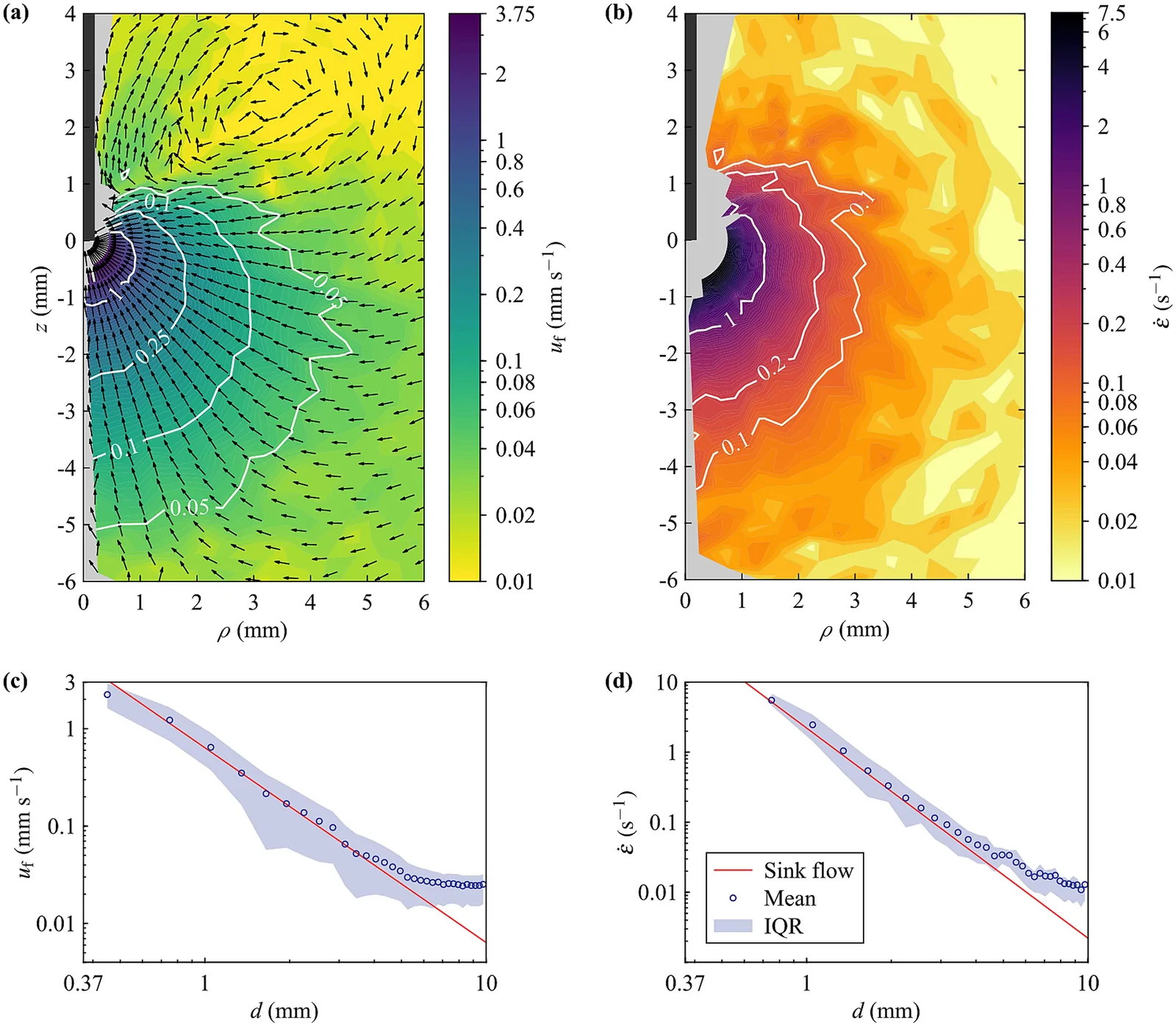
Spatial map of the (a) flow speed, *u*_f_, (color) and direction (unit vectors) and (b) strain rate, $\dot{\varepsilon}$, in the *ρ*-*z*-plane. Grey areas indicate regions with missing data. Mean values and interquartile ranges (IQR) of (c) *u*_f_ and (d) $\dot{\varepsilon}$ as a function of the distance to needle tip, *d*, compared to a theoretical sink flow. Dispersion is shown as the IQR to reflect skew and robustness to outliers.

The local Reynolds number, Re_f_, was consistently below 1.0 across the flow field, with a maximum of 0.8 near the needle tip, indicating creeping-flow conditions. Evaluation of *Q* confirmed a strain-dominated flow near the tip (*Q* < 0) and negligible gradients in the far field (*Q* ≈ 0) (Fig. S1). Therefore, subsequent quantitative analyses of copepod behavior focused on local flow speed *u*_f_ and strain rate $\dot{\varepsilon}$.

### Copepod behavior at no-flow condition

In the no-flow condition (**u**_f_ = 0), of the 90 trajectories, the closest approach reached a minimum distance to the needle tip *d*_min_ ≈ 1.6 mm, whereas all others had *d*_min_ > 2.6 mm. One individual settled near the upper end of the needle and scuttled in that region. Apart from this case, copepods exhibited two primary locomotion modes: (i) hopping and (ii) sinking. Hopping consisted of rapid, discrete bursts of active propulsion, predominantly directed vertically upward to counter gravity, but also occurring at various angles. Between jumps, copepods sank, with sink phases ranging from brief pauses to prolonged passive descents. Across trajectories, the cumulative duration of jumps accounted for ~42% of total time. A video of a representative test run illustrating the two locomotion modes is provided in the Supplementary Data (Video NF), with synchronized camera recordings and reconstructed 3D trajectories.

Jump intensity was *u*_jump_ = 36.7 ± 7.8 mm s^−1^ (mean ± SD), with a maximum of 56.2 mm s^−1^. The separation time between successive jumps was *T*_jump_ = 0.21 ± 0.15 s. Jump distance was *d*_jump_ = 1.4 ± 0.6 mm, and jump duration was Δ*t*_jump_ = 0.16 ± 0.04 s.

Frequency distributions of the radial, *u*_rel,*ρ*_, azimuthal, *u*_rel,*ϕ*_ and vertical, *u*_rel,*z*_, components of the relative velocity showed concentration around zero, indicating a high frequency of low values (orange lines in Fig. 3a–c). All three velocity components were within approximately ±50 mm s^−1^. The distributions of *u*_rel,*ρ*_ (Fig. 3a) and *u*_rel,*ϕ*_ (Fig. 3b) were symmetric with sharp central peaks at zero. In contrast, the distribution of *u*_rel,*z*_ peaked at −2.3 mm s^−1^, matching the sinking speed *u*_sink_ = 2.3 ± 0.6 mm s^−1^, and exhibited a larger proportion of upward motions (*u*_rel,*z*_ > 0) attributable to jumps against gravity (Fig. 3c).

**Fig. 3:**
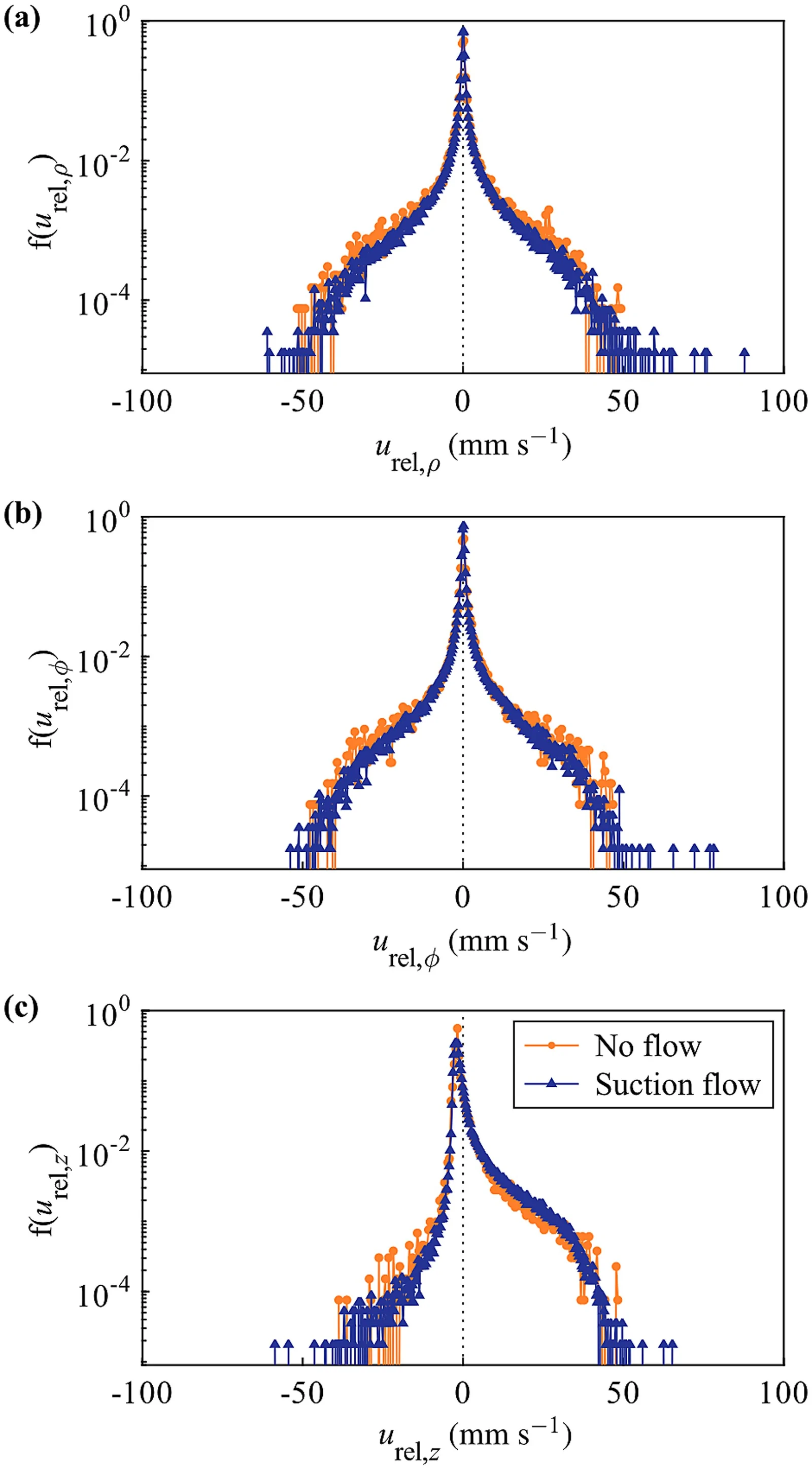
Frequency distributions of the (a) radial, *u*_rel,*ρ*_, (b) azimuthal, *u*_rel,*ϕ*_, and (c) vertical, *u*_rel,*z*_, components of *u*_rel_ for both the no-flow and suction-flow conditions.

### Behavioral responses to suction flow

In the presence of suction flow, the frequency distributions of the relative-velocity components did not differ substantially from those under the no-flow condition, although rare escape jumps attained higher peak speeds (blue lines in Fig. 3a–c). Likewise, jump metrics were comparable: *u*_jump_ = 33.3 ± 9.4 mm s^−1^, *T*_jump_ = 0.24 ± 0.28 s, *d*_jump_ = 1.3 ± 0.7 mm and Δ*t*_jump_ = 0.16 ± 0.04 s. The cumulative duration of jumps accounted for ~41% of the total observation time.

Mean values of the copepod’s absolute and relative velocity components along **d**, *u*_c,*d*_ and *u*_rel,*d*_, were approximately zero for *d* > 3 mm (Fig. 4a). Closer to the needle tip, the mean *u*_rel,*d*_ increased toward the tip, reaching a maximum of 1.2 mm s^−1^ and effectively compensating the mean flow velocity component *u*_f,*d*_, such that *u*_c,*d*_(*d*) ≈ 0. The mean movement direction was approximately tangential to the tip (*θ* ≈ 90°) for *d* > 1 mm (Fig. 4b). Only very near the tip (*d* < 1 mm) was there an overall tendency to move away from it (*θ* > 90°), coinciding with a slightly higher probability *P*(*u*_rel,*d*_ > 0) of 60% within 2 mm of the tip (Fig. 4c). At larger distances, *P*(*u*_rel,*d*_ > 0) ≈ 0.5, consistent with a fair (symmetric) Bernoulli model for the sign of *u*_rel,*d*_.

**Fig. 4:**
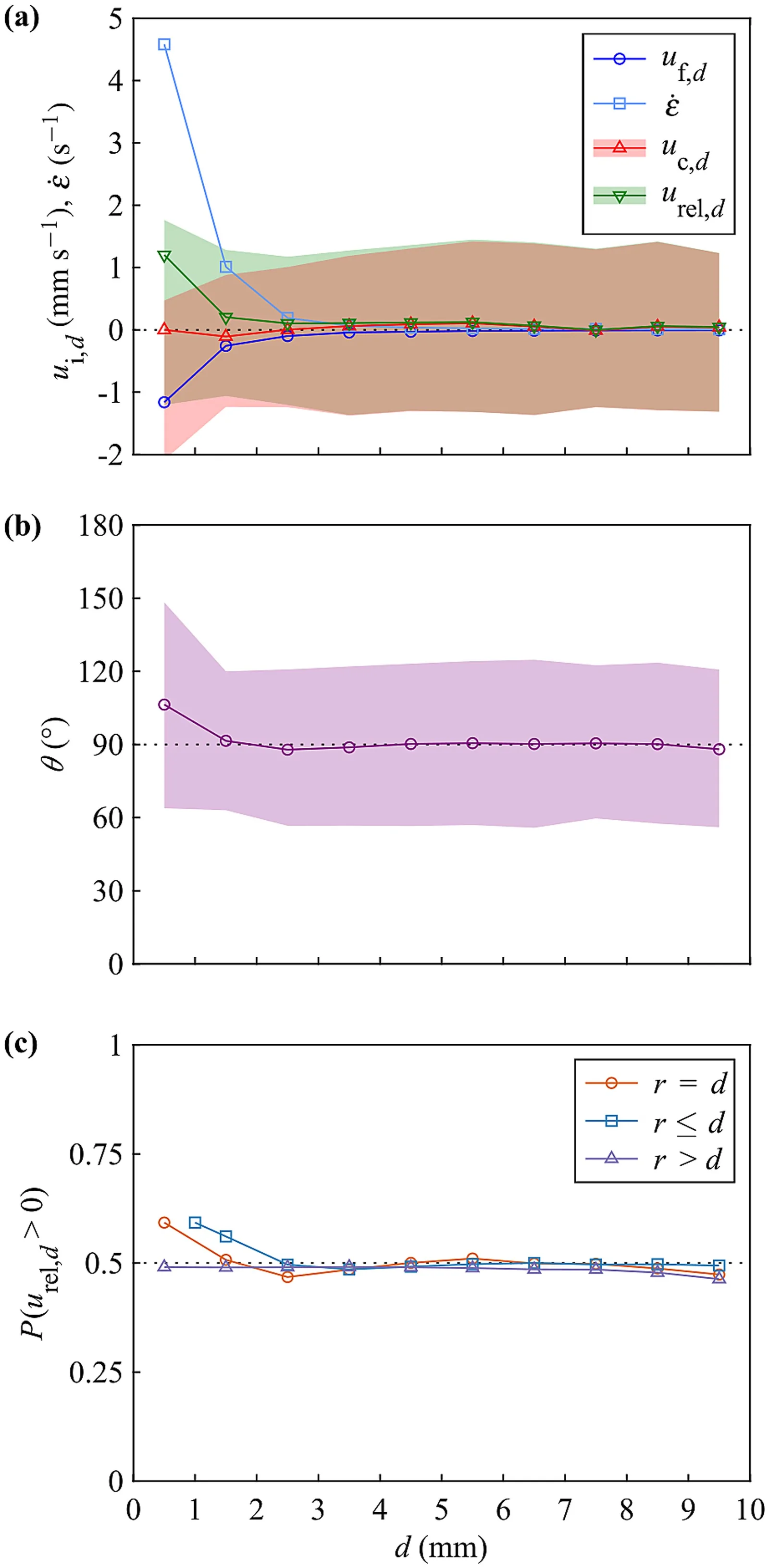
Variations of (a) the average flow, *u*_f,*d*_, copepod, *u*_c,*d*_, and relative, *u*_rel,*d*_, velocity components along **d**, together with the strain rate $\dot{\varepsilon}$; (b) the average angle *θ* between **u**_rel_ and **d**; and (c) the probability of *u*_rel,*d*_ > 0 for different ranges of radial distance *r*, all as functions of the distance to the needle tip, *d*. Shaded bands indicate interquartile ranges, reflecting skew and robustness to outliers.

While hopping and sinking remained the predominant locomotion modes under suction flow, some copepods approaching the needle tip executed one or two distinct escape jumps, characterized by a sudden reorientation away from the tip (Fig. 5c) and substantially higher intensity than typical hops (Fig. 5b). Tracking data and video observations showed that all escape events were initiated within 1.8 mm of the needle tip, and all but one (E4) were initiated within 0.8 mm (Fig. 5a). Among individuals that approached within *d*_min_ < 1.8 mm, 8 of 25 (32%) exhibited at least one escape jump. Corresponding videos are provided in the Supplementary Data (Videos E1–E8), with synchronized camera recordings and reconstructed 3D trajectories. In all analyzed encounters, escape jumps were initiated immediately after copepods reached the absolute minimum distance to the tip over the entire track, *d*_min_. The corresponding time, *t*(*d*_min_), was taken as the escape initiation (index “e”). Local hydrodynamic conditions at *t*(*d*_min_) averaged *u*_rel,e_ = 6.4 ± 4.2 mm s^−1^ and $\dot{\varepsilon}$_e_ = 4.7 ± 2.0 s^−1^.

**Fig. 5:**
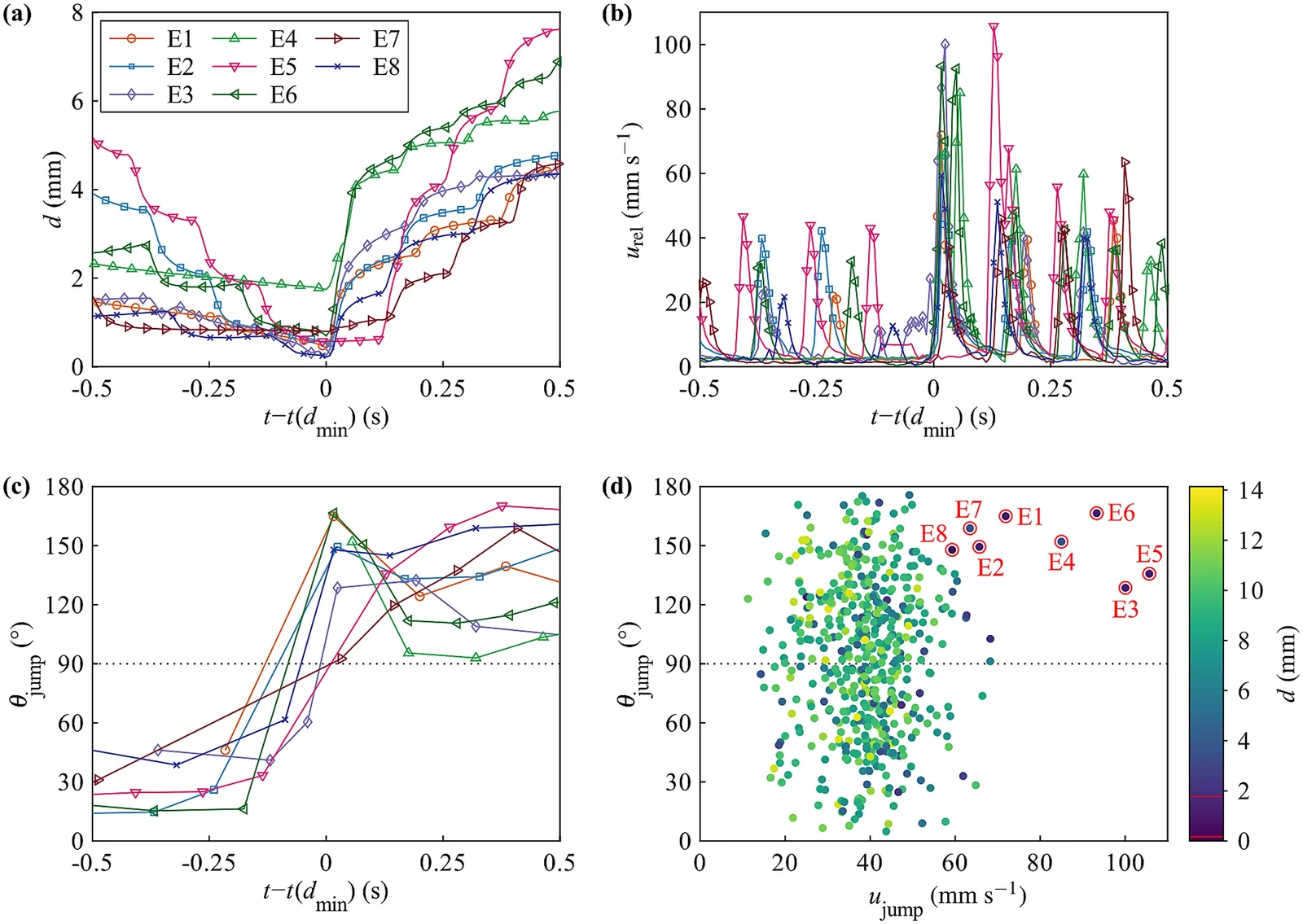
Variations of (a) distance, *d*, (b) relative speed, *u*_rel_, and (c) jump direction, *θ*_jump_, over time *t*, around the time of minimum distance to the needle tip, *t*(*d*_min_), for the eight trajectories exhibiting escape reactions (E1–E8). (d) Jump direction, *θ*_jump_, as a function of jump intensity, *u*_jump_, for the strongest jump of each trajectory. Circled points indicate the eight escape jumps.

Escape-jump intensities averaged *u*_jump,e_ = 80.6 ± 17.9 mm s^−1^, with a maximum of 105.7 mm s^−1^, while the maximum intensity of a regular hop was 68.3 mm s^−1^ (Fig. 5b). Before reaching *d*_min_, jumps were predominantly oriented toward the needle tip (*θ*_jump_ < 90°). Within 0.5 s after *t*(*d*_min_), jumps were prevalently oriented away from the tip, i.e. *θ*_jump_ > 90° (Fig. 5c). All identified escape jumps had *u*_jump,e_ > 59 mm s^−1^, exceeding the 99.5th percentile of all jump intensities. Comparing the maximum-intensity jump from each trajectory, all eight escape events were oriented away from the tip (*θ*_jump,e_ > 90°) and showed a trend toward higher *u*_jump_ relative to other trajectories (Fig. 5d).

As in the no-flow treatment, a few individuals settled on and scuttled along the needle surface without exhibiting an escape response. Moreover, in five of the eight identified escape events (E1–E3, E5, E8), individuals appeared to be close enough to the needle tip that they may have contacted it with their bodies or appendages before jumping (Fig. 6).

**Fig. 6:**
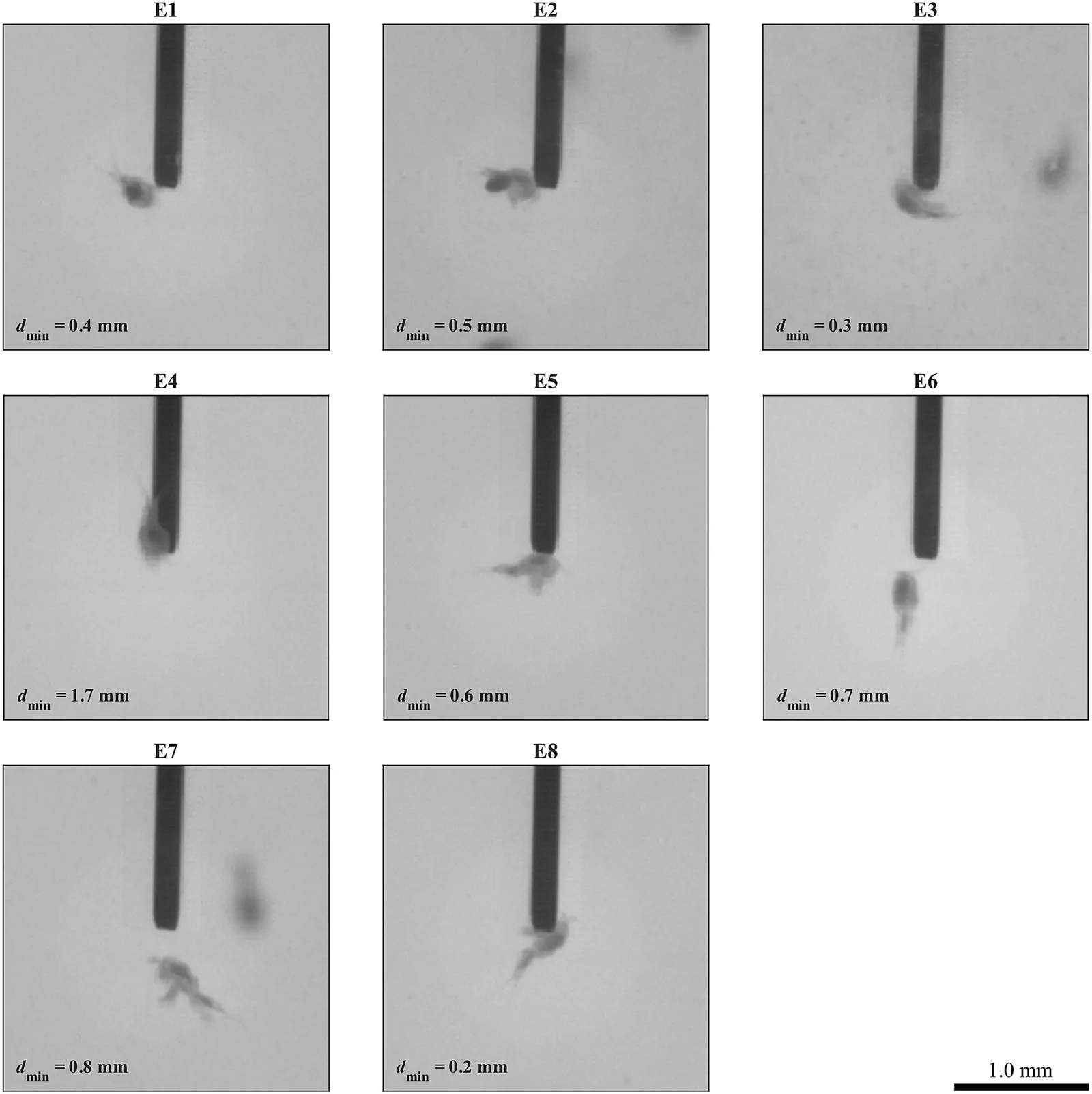
Front-facing camera frames at the instant of minimum distance to the needle tip, *t*(*d*_min_), for the eight identified escape reactions (E1–E8).

## DISCUSSION

Copepod behavioral ecology has been dominated by pelagic-focused studies, leaving benthic taxa comparatively understudied. We examined the behavior of the benthic harpacticoid *T. californicus* under no-flow conditions and in the presence of a localized suction flow, for the first time to our knowledge. Prior work using siphon-generated suction has focused predominantly on predator-like stimuli, identifying thresholds in flow speed and/or velocity-gradient components that evoke escape (Table I). Those studies typically employed stronger suction or larger inlets, producing higher far-field velocities and broader deformation fields. Here, we deliberately used a thin needle operated at a low flow rate to create a controlled suction flow with weak far-field gradients and a steep intensification near the tip. This idealized stimulus allowed us to examine, within a single well-characterized flow, routine hop-and-sink under weak background gradients and high-intensity escapes near the tip, both largely undocumented for *T. californicus*. Copepod trajectories and the suction flow field were quantified by 3D PTV at a scale and level of detail that has been rarely achieved in previous investigations (Burdick *et al.,* 2007; Yen *et al.,* 2015).

Our experiments showed that—consistent with other harpacticoids (Armonies, 1988; Hwang and Turner, 1995)—*T. californicus* is not strictly bottom-dwelling. Individuals frequently entered the water column and actively regulated vertical position using a characteristic hop-and-sink gait. During this locomotion, sinking occupied ~60% of total time, while jumping accounted for 40%, and peak routine jump intensities reached up to 68 mm s^−1^. Although hop-and-sink locomotion is well documented in calanoids (Tiselius and Jonsson, 1990), it has been rarely described for harpacticoids (Hwang and Turner, 1995). Given that many harpacticoids are primarily grazers (De Troch *et al.,* 2006), excursions into the water column may facilitate active changes in position (Azovsky and Udalov, 2026), but this remains a hypothesis beyond the scope of our study.

In the presence of suction flow, overall kinematic statistics (jump intensity, distance, duration and frequency) were indistinguishable from no-flow controls. At distances *d* > 2 mm from the needle tip, orientation statistics were consistent with random swimming and showed no attraction to or avoidance of the source. Upon entering the near field of the stimulus (*d* < 2 mm)—where flow intensities may mimic predator-like signals—copepods exhibited active movement against the flow, compensating advection and thereby avoiding suction into the needle. Within 1.8 mm of the tip, 8 of 25 individuals (32%) executed one or two powerful, directed escape jumps away from the source after reaching minimum distance. Prior work on other species has documented similar escape responses, including one or several consecutive “long jumps” or “thrusts” that displaced copepods out of high-velocity zones (Kiørboe *et al.,* 1999; Waggett and Buskey, 2007).

In five of the eight identified escape events, individuals may have contacted the needle tip with their body or appendages immediately prior to the evasive manoeuvre. At the same time, several individuals settled on and scuttled along the needle surface in both no-flow and suction-flow treatments without escaping, indicating that contact per se does not elicit an escape. Notably, three escape events occurred without apparent contact, consistent with a response to hydrodynamic disturbance. To our knowledge, there is no clear evidence documenting escape responses in copepods triggered solely by direct tactile stimuli. Furthermore, given its benthic lifestyle, routine contact with surfaces is expected for *T. californicus*. Consequently, we hypothesize that the flow disturbance was the main trigger for escape, although we cannot completely rule out a contact contribution. A larger sample of unequivocally non-contact escape events will be required to rigorously test this hypothesis.

Because direct contact cannot explain all escape events, the distance-dependent flow kinematics indicate a localized trigger tied to the increasing disturbance intensity as copepods approached the tip, consistent with previous studies on other species (Kiørboe *et al.,* 1999; Gilbert and Buskey, 2005; Waggett and Buskey, 2007). Threshold strain rates at escape onset, $\dot{\varepsilon}$_e_, were comparable to values reported for calanoids, whereas relative speeds, *u*_rel,e_, were higher (Table I). However, we infer that escapes are triggered by fluid strain rate, or closely related gradient metrics, rather than by flow speed per se, because only fluid-body relative motion can be sensed and relative flow-speed magnitudes observed at escape initiation were frequently exceeded during routine hops without eliciting evasive manoeuvres. Interpreting fluid deformation as the principal sensory cue aligns with mechanosensory principles established for pelagic taxa (Kiørboe *et al.,* 1999; Tuttle *et al.,* 2019). However, assessing their transferability to *T. californicus* and benthic species more generally will require experiments that decouple relative speed from fluid deformation by using stimuli with matched velocity magnitudes but differing gradients (e.g. microjets versus sinks).

Despite its primarily benthic habit and morphology distinct from pelagic taxa, *T. californicus* can perform directed, powerful escape jumps in response to hydrodynamic stimuli. However, peak escape speeds were considerably lower than those reported for pelagic taxa (Table I). This comparatively lower escape capacity in the water column may reflect the species’ benthic-oriented morphology. Nevertheless, individuals approximately doubled their jump intensity during escapes relative to routine hopping.

These results indicate a context-dependent modulation of locomotion: *T. californicus* maintains a routine hop-and-sink gait under weak background deformation and switches to high-intensity escape manoeuvres when encountering a localized, more intense flow disturbance (e.g. induced by a predator). Our findings indicate that the benthic specialization of *T. californicus*—and potentially other harpacticoids—does not preclude high-performance evasion.

We characterized the behavior of a single benthic species exposed to a steady, localized suction flow under quiescent conditions and observed a modest number of clear escape events. Replication across additional non-pelagic taxa, life stages and a broader range of flow intensities will be essential to generalize statements about routine locomotion in benthic copepods and their sensitivity to local flow disturbances. Such work would also enable direct comparison with previously derived thresholds for calanoids (Table I) on a common gradient-based scale.

In nature, benthic habitats are structurally complex, with rough substrates, vegetation and unsteady wave-current boundary layers (Robinson *et al.,* 2007). Future work should incorporate these features to evaluate how flow heterogeneity and confinement shape general behavior and responses to localized flow signals, including disturbances generated by nearby conspecifics and other organisms.

## CONCLUSIONS

We quantified the locomotion of the benthic harpacticoid *T. californicus* in still water and under localized suction flow using synchronized camera recordings, 3D trajectory reconstruction and PTV. Individuals frequently entered the water column and exhibited a routine hop-and-sink gait in both conditions. Suction did not substantially alter routine kinematics in the far field relative to no-flow conditions but elicited rapid escape manoeuvres with higher peak speeds in the near field, consistent with an evasive repertoire associated with localized flow gradients. Contrary to our hypothesis, threshold strain rates at escape onset were comparable to values reported for calanoids, indicating similar sensitivities to predator-like hydrodynamic disturbances. However, as hypothesized, peak escape speeds were considerably lower than those reported for pelagic taxa, presumably due to the species’ primarily benthic habit and morphology.

This study advances the understanding of mechanosensation in a benthic copepod species. Curated datasets (smoothed trajectories for no-flow and suction-flow trials) and illustrative videos accompany the present study to support reuse, benchmarking and model development. Our experiments were conducted in a controlled laboratory microenvironment on a single species and life stage. Therefore, generalization to other taxa, flow geometries and environmental contexts should be made cautiously. Future work should further quantify the flow thresholds and spatial gradients that trigger escape, disentangle tactile from flow cues and test whether these patterns persist in more complex, habitat-relevant settings.

## Supplementary Material

Supplementary_fbag080

## Data Availability

The data that support the findings of this study are openly available in *Zenodo* at http://doi.org/10.5281/zenodo.18846098.

## Notes

Corresponding editor: Hans Dam
